# Acceptability of Intervention Design Factors in mHealth Intervention Research: Experimental Factorial Study

**DOI:** 10.2196/23303

**Published:** 2021-07-26

**Authors:** Frank T Materia, Joshua M Smyth

**Affiliations:** 1 Division of Health Services and Outcomes Research Children's Mercy Kansas City Kansas City, MO United States; 2 Departments of Biobehavioral Health and Medicine The Pennsylvania State University University Park, PA United States

**Keywords:** mHealth, acceptability, implementation, health behavior, smartphone, mobile phone, wearable

## Abstract

**Background:**

With the growing interest in mobile health (mHealth), behavioral medicine researchers are increasingly conducting intervention studies that use mobile technology (eg, to support healthy behavior change). Such studies’ scientific premises are often sound, yet there is a dearth of implementational data on which to base mHealth research methodologies. Notably, mHealth approaches must be designed to be acceptable to research participants to support meaningful engagement, but little empirical data about design factors influencing acceptability in such studies exist.

**Objective:**

This study aims to evaluate the impact of two common design factors in mHealth intervention research—requiring multiple devices (eg, a study smartphone and wrist sensor) relative to requiring a single device and providing individually tailored feedback as opposed to generic content—on reported participant acceptability.

**Methods:**

A diverse US adult convenience sample (female: 104/255, 40.8%; White: 208/255, 81.6%; aged 18-74 years) was recruited to complete a web-based experiment. A 2×2 factorial design (number of devices×nature of feedback) was used. A learning module explaining the necessary concepts (eg, behavior change interventions, acceptability, and tailored content) was presented, followed by four vignettes (representing each factorial cell) that were presented to participants in a random order. The vignettes each described a hypothetical mHealth intervention study featuring different combinations of the two design factors (requiring a single device vs multiple devices and providing tailored vs generic content). Participants rated acceptability dimensions (interest, benefit, enjoyment, utility, confidence, difficulty, and overall likelihood of participating) for each study presented.

**Results:**

Reported interest, benefit, enjoyment, confidence in completing study requirements, and perceived utility were each significantly higher for studies featuring tailored (vs generic) content, and the overall estimate of the likelihood of participation was significantly higher. Ratings of interest, benefit, and perceived utility were significantly higher for studies requiring multiple devices (vs a single device); however, multiple device studies also had significantly lower ratings of confidence in completing study requirements, and participation was seen as more difficult and was associated with a lower estimated likelihood of participation. The two factors did not exhibit any evidence of statistical interactions in any of the outcomes tested.

**Conclusions:**

The results suggest that potential research participants are sensitive to mHealth design factors. These mHealth intervention design factors may be important for initial perceptions of acceptability (in research or clinical settings). This, in turn, may be associated with participant (eg, self) selection processes, differential compliance with study or treatment processes, or retention over time.

## Introduction

### Background

Most health behavior change programs have historically required participants to attend in-person appointments or sessions, with a trained clinician or facilitator guiding intervention delivery. However, in-person delivery modes can be costly, time consuming, and burdensome for both participants and providers. The field of mobile health (mHealth) broadly examines how portable, wireless technologies (eg, smartphones, wearable fitness trackers, and smartwatches) can be used in an effort to reduce costs, enhance access, and increase the reach of health behavior change interventions. The ubiquity of mobile technology ownership across populations and contexts makes mHealth uniquely positioned to supplement or replace behavior change programs traditionally delivered in person [1].

### mHealth Design and Implementation Science

The field of mHealth is still relatively new as a scientific discipline (ie, approximately 2 decades old) [2]. Recent literature has demonstrated the efficacy of some mHealth approaches for improving behavioral health outcomes [3-5]. A key factor in mHealth implementation underlying potential efficacy is the willingness and capability of users (eg, patients and research participants) to successfully engage with the mHealth delivery system. There is still very little research formally examining evidence-based methods for designing mHealth to support uptake and adherence with end users (eg, participants enrolled in a technology-based behavior change program) or, conversely, what study design features may be inhibiting engagement (particularly *early on* such as at initial study enrollment). This issue is particularly vital in mHealth research, given the large number of design factors and considerations in play. Some common design decisions mHealth intervention researchers must consider, among many, including selecting which technologies (eg, smartphone vs tablet) will be used to deliver intervention content, determining how to deliver intervention messaging (eg, text vs videos), deciding on the style of messaging (eg, personalized vs generic content), and requiring persistent internet connectivity (ie, if internet connectivity is necessary to push content and share data or if content and data stay native to the device).

Rigorous research evaluating how different design decisions may enhance or hinder end user engagement with mHealth is limited. One possible solution for filling these gaps is integrating research approaches from implementation science. The goal of implementation science is to evaluate methods for integrating evidence-based supports (eg, mHealth) into practice, with the goal of enhancing the successful delivery and effectiveness of new (or underutilized) behavior change approaches [6]. One key aim of implementation science involves evaluating different intervention design features (eg, varying choices for delivery modes and content selection) and understanding how they affect successful program implementation (eg, support uptake and adherence to the intervention among participants [7]).

### Acceptability in mHealth Design and Implementation

One implementation factor directly related to the intervention design is participant *acceptability*. One broad definition of acceptability is “the quality of being tolerated or allowed” [8], and the other is “the quality or state of meeting one’s needs adequately” [9]. In terms of mHealth, one proposed definition of acceptability is “an end-user’s subjective perceptions of, and measurable sustained engagement with, a mobile health intervention system...(including) perceived satisfaction, willingness, and agreeability” [10].

The premise of studying acceptability as part of mHealth implementation research is rooted in the theory of behavioral science. For example, the theory of planned behavior (TPB) explains how individuals’ beliefs are associated with their actions [11]. The TPB proposes that individuals’ motivations to adopt certain behaviors are driven by behavioral (eg, advantages to behavior change), normative (eg, external expectations), and control (eg, barriers to behavior change) beliefs. Tenets of the TPB can be applied to inform the study of acceptability in mHealth, especially for informing which dimensions of acceptability are important to evaluate. As the TPB proposes, there are multiple psychological factors that influence behavioral adoption. Similarly, acceptability is a multidimensional construct that includes perceptions of interest (piques participant curiosity), enjoyment (participation is pleasurable), difficulty (how tough participation will be), utility (individual value in participating), benefit (participation is advantageous), and confidence (perceived ability to accomplish participation) [10,12,13]. Researchers evaluating these dimensions can determine which mHealth intervention design elements are preferred by participants and which may limit motivation for participation. Such research allows for a better understanding of participant selection processes, differential compliance, or adherence over time in mHealth intervention research, which are factors related to effective and meaningful engagement with behavior change interventions [13-15].

Acceptability can also be assessed at different time points in the user experience—a priori and posteriori. Evaluating a priori acceptability (eg, *pre* or *prospective* acceptability) involves assessing end user acceptability for the design of a system before actually engaging with it. An a priori assessment of acceptability is particularly useful during the intervention design process, as it provides researchers with proxy ratings for how design decisions may influence individuals’ initial beliefs, perceptions, motivation, and likelihood of uptake and adherence to program requirements in daily life (eg, participation in a research study). To assess a priori acceptability, researchers typically introduce a description or prototype of the intervention to the participant and evaluate their perceived acceptability of the intervention’s various requirements. This is most commonly measured via self-report surveys and measures or through semistructured focus group interviews [12-14].

Posteriori acceptability (ie, *post* or *retrospective* acceptability) is evaluated after an end user has been introduced to a system and they have spent time engaging with it [14,15]. As opposed to a priori assessment (which is hypothetical and only predictive of intervention uptake), a posteriori assessment can inform researchers of how acceptability may change over the course of intervention participation. Posteriori acceptability can also serve as a measure of ongoing participant engagement and buy-in with the program [14,15].

### Limitations in Evaluating Acceptability in mHealth Design

Given the recent proliferation of mHealth technologies worldwide (eg, smartphones and wearables [1,16]), researchers from multiple disciplines (eg, behavioral medicine and computer science) have expressed an imperative need to evaluate acceptability as part of the mHealth intervention design process [17-22]. Acceptability information informs design decisions that may enhance participant motivation to meaningfully engage with the technology and may positively impact their likelihood of adopting effective behavioral change [2,23]. However, despite the recognition of a need, research specifically focused on evaluating how different design decisions influence mHealth intervention acceptability is limited. The studies that have been conducted have mostly used small, homogenous samples with limited generalizability [24]. Most design studies have also been descriptive (eg, using focus groups or brief surveys to evaluate acceptability) and lacked experimental designs. Experimental methods (eg, randomization and manipulation of technology design features) can be particularly informative for studying a priori acceptability because they afford controls that allow researchers to assess whether significant differences in acceptability ratings before participating in a mobile intervention are because of varying conditions of mHealth design factors [25,26]. Surprisingly, many mHealth design studies to date have also lacked attention to theory from implementation and behavioral sciences to inform evaluation plans [27]. The literature is unclear regarding how various mHealth design conditions differentially influence participant acceptance of the intervention and its participation requirements [10,28].

### Common mHealth Design Features

To experimentally evaluate how mHealth design components influence acceptability (specifically, a priori acceptability), an approach was taken to identify a small number of representative design features to study (from the large array of possible design options), which allows for the examination of several intervention features that are already extensively used in mHealth and also serves as an example (*proof of concept*) for this method more generally (that may be applicable to other design features as well). Two mHealth intervention methods of interest are requiring a single mobile device rather than multiple devices (eg, for broader data collection or alternate modes of content delivery) and providing tailored intervention content to individual participants (rather than providing the same generic content to all participants).

An advantage of requiring participants to use multiple devices (eg, smartphones, smartwatches, and ambulatory heart rate monitors) in a study is that researchers can obtain diverse data streams and use a variety of interfaces to deliver content [29-31]. A commonsense worry about this approach, partially supported by previous posteriori research, is that end users report feeling overwhelmed and exhibit lower acceptability after participating in a program that requires the concurrent use of multiple devices [32]. However, research examining a priori acceptability for participating in an mHealth program that requires multiple devices is less clear. If requiring multiple devices reduces a priori acceptability, participants may be less likely to participate, but research examining this is scant.

Although much intervention content is standardized and delivered generically to all participants, there is growing interest in providing intervention elements tailored for each participant. In brief, this involves providing content that is specific to each individual end user (eg, prompting participants by name and generating messaging based on person-specific health indicators). Previous work suggests that tailoring may have beneficial effects on intervention outcomes [1,33,34]. In addition, previous work measuring posteriori acceptability has demonstrated that participants positively perceive tailored content as enhancing their satisfaction with behavioral interventions; however, little empirical work has focused on investigating how this provision may influence a priori acceptability [33,35].

There is no existing research that has specifically examined the dimensions of a priori acceptability for participating in an mHealth intervention that requires multiple device utilization and provides tailored intervention content. Previous posteriori studies have examined these two factors separately after intervention engagement and found that they are meaningful for acceptability after participation is complete, but there is little research focused on understanding how potential mHealth participants may respond to such design components in the early stages of recruitment, as the a priori acceptability of device requirements and tailoring may influence individuals’ motivation for selecting to participate in mHealth research. Furthermore, given the general lack of experimental research in this space, the aspects of such designs that are reliably related to positive perceptions of a priori acceptability are largely unknown (past observational inference). These are important factors for researchers to understand and consider in designing mHealth interventions to support participant motivation, uptake, engagement, and adherence to program requirements. More generally, this method serves as proof of concept for how other design features can be tested—singly or in combinations of factors—and using assessments of a priori acceptability, posteriori acceptability, or both.

### This Study

This study sought to understand the impact that two common design decisions in mHealth behavior change interventions—requiring a single versus multiple devices (a study smartphone and a wrist sensor) and providing tailored as opposed to generic content—have on a priori participant acceptability. Specifically, the goal was to understand if participants were sensitive to changes in factors of these mHealth design decisions and how these changes may affect various dimensions of a priori acceptability, which act as proxy indicators of participant motivation and likelihood for meaningful engagement with mHealth intervention content and requirements. Previous research and theory from behavioral science (ie, TPB) informed the design of the study instrument and the dimensions of acceptability measured (ie, perceived interest, benefit, utility, enjoyment, confidence and difficulty in participating, and perceived likelihood of participating [12,13,36]). This was an experimental 2×2 factorial design study using cross-sectional web-based survey methods. One factor was the number of devices required (1 vs 2), and the second factor was the nature of feedback (personalized vs generic); thus, each cell represents a different mHealth study scenario that featured different combinations of the two mHealth design factors of the study. As each participant received all four scenarios, the presentation order of the study vignettes was randomized to alleviate possible ordering and conditioning effects.

## Methods

### Recruitment and Study Design

An experimental 2×2 factorial web-based study was conducted in September 2018. Participants were recruited via Amazon Mechanical Turk (MTurk), a web-based crowdsourcing platform. MTurk provides a portal through which participants (ie, US citizens aged >18 years) find paid web-based research study opportunities. Participants have dedicated MTurk profiles that contain their demographic information (eg, age, gender, and income). Researchers can indicate specific inclusion and exclusion criteria for their MTurk study and advertise the study on MTurk, and participants are able to choose to participate in studies that interest them in MTurk and also indicate their desired sample size for the study and provide Amazon directly with a payment for all participants’ compensation. Participants were provided compensation on completion of the study’s survey; payment was transferred directly via Amazon to the participants’ preferred accounts.

When verified, eligible participants log in to their MTurk account on the web, and they see a list of eligible survey and study opportunities or human interaction tasks (HITs). The compensation provided for completing the study is listed, along with the approximate length of time that the HIT will take to complete, and a brief description of the study. Participants were free to choose the HIT that they were interested in and eligible for from the list.

The name of this study’s HIT was *The SmartHealth Preferences Study*. The HIT indicated that the study takes approximately 12 minutes to complete, and participants were paid US $5.00 to complete the entire study. Once they clicked on the HIT in MTurk, participants were taken directly to the study, and informed consent was obtained. The study was hosted on Qualtrics’ CoreXM (SAP SE) web-based survey platform via a secure university account.

After obtaining informed consent, participants were presented with a learning module that ensured that they were familiar with the relevant concepts and terminology necessary for completing the study. The learning module defined, in lay terms, a health behavior, specifically, “any action that a person takes that affects their health.” Examples included smoking cigarettes—BAD health behavior, healthy eating—GOOD health behavior, binge drinking alcohol—BAD health behavior, staying fit and active—GOOD health behavior. Then, a broad definition of a health behavior change intervention program was described, that is, “any planned series of events, like classes, routines, or group meetings, that aims to encourage its participants to live healthier lifestyles and make healthier decisions for themselves.” The example provided described how Jenny Craig and WeightWatchers are health behavior change programs aimed at promoting nutritional and physical health to help participants with weight loss. This was followed by an explanation of what a mobile device is, that is, “examples [of a mobile device] include a smartphone or mobile phone, FitBit or smartwatch, or iPad or tablet computer.” A *single device* was described as only needing to carry or use one mobile device (eg, a smartphone). *Multiple devices* was described as needing to carry or use more than one mobile device (eg, a smartphone plus wear a smartwatch). Then, the learning module explained the difference between generic versus personalized program messages, broadly using physical activity as an exemplar behavior for which these intervention messages could be delivered to support. Generic (ie, nontailored) messages were described as, “when all participants in a health behavior change program receive the same messages and notifications.” An example included a generic message from a fitness program: “Keep up the good work staying active, you can do it!” Personalized (ie, tailored) messages were described as, “when participants in a health behavior change program receive messages and notifications that are specific to them and their personal goals.” An example provided demonstrated a personalized message from a fitness program: “Mary, you walked 10,500 steps today—you beat your personal goal! Keep up the good work staying active!”

**Figure 1:**
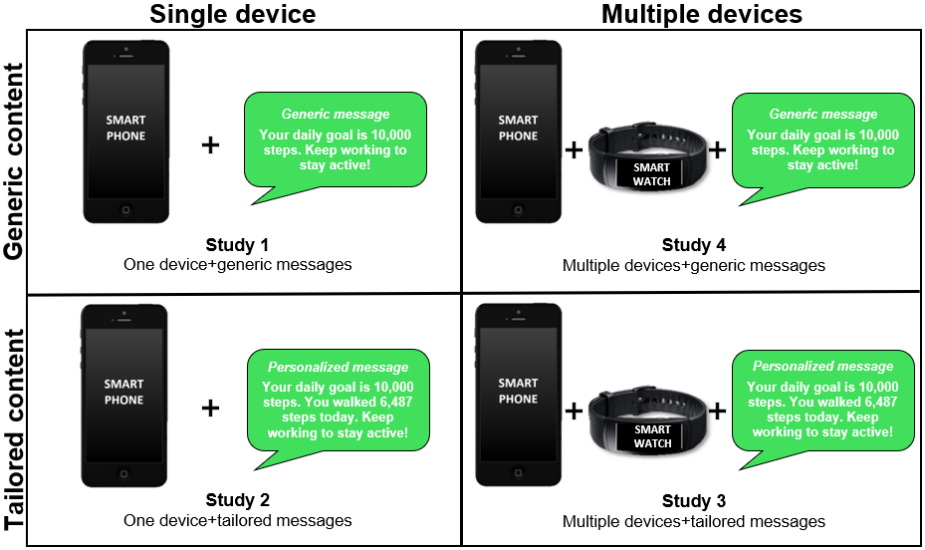
A 2×2 factorial design exploring multiple dimensions of acceptability for mobile health study scenarios featuring different combinations of a single device versus multiple devices and tailored versus generic content.

As additional examples to visually reinforce and remind participants about the differences between single versus multiple device requirements and generic versus personalized content, participants were shown various graphics. A graphic of just a smartphone was presented to indicate the meaning of *single device* and a graphic depicting a smartphone, plus sign (+), and then a coupled smartwatch was presented to indicate the meaning of *multiple devices*. Two graphics designed to look like a text message notification on a smartphone were displayed to further reinforce the concept of generic versus tailored (ie, personalized) content. Participants were able to read examples of both types of content in the graphics. The generic message picture example read, “Your daily goal is 10,000 steps. Keep working to stay active!” indicating a nontailored message. The personalized message picture example read, “Your daily goal is 10,000 steps. You walked 6,487 steps today. Keep working to stay active!” indicating a tailored message. Figure 1 shows the graphics shown to the participants to reinforce these concepts.

After completing the learning modules, participants advanced to a new screen in the survey, where they were instructed to imagine themselves being involved in a health behavior change intervention. They were told that they would be presented with different program scenarios where requirements for the number of devices and whether they receive generic or personalized content would be different. Then, four vignettes describing each cell of a 2×2 factorial were delivered in a random sequence for each participant. Hypothetical mHealth intervention studies were described in each vignette, featuring varying combinations of the design factors being studied. Vignette 1 described an mHealth study scenario requiring a single device and provided generic content, vignette 2 was a single device with tailored content, vignette 3 required multiple devices and provided tailored content, and vignette 4 required multiple devices and provided generic content. Each vignette also displayed appropriate graphics from the earlier learning module that were provided on the screen for each hypothetical study scenario to further ensure participants understood the combination of tailoring and device factors featured in the vignette on their computer screen (Figure 1).

### Measures

At the end of each mHealth design vignette, participants were asked to rate five dimensions of their a priori acceptability for participating in that particular mHealth design scenario. The specific dimensions chosen for assessment (ie, interest, benefit, enjoyment, utility, confidence, and difficulty) were based on constructs from the TPB (ie, behavioral intentions and behavioral control) as well as previous work assessing behavioral motivation and a priori mHealth design preferences [12]. Participants rated each acceptability dimension using a 5-point Likert scale (eg, “How interesting would participating in this study be?”; 1=not interesting; 5=very interesting). Participants also rated their overall likelihood of participating in the study on a 100-point visual analog slider scale that displayed their indicated value (ie, “if given the chance, how likely would you be to participate in this study?” 0=not at all likely to 100=extremely likely), which was broadly based on the TPB’s construct of intentions (ie, intended likelihood of participating) predicting behavioral engagement (ie, actual participation in the study) [10,12].

Attention checks were also administered to assess data quality and participant comprehension while completing the web-based study. Five careless responder questions were included in the study. These questions asked participants to indicate information that they should have read, had they been paying attention (and not simply clicking through the questions). Using careless responder items in web-based survey research has been shown to be an effective method for confirming the reliability and accuracy of participant data [37]. After reading each vignette, the participant was presented with a careless responder question that asked them to confirm what they just reviewed. For example, after reading the vignette description for the *single device and generic content* scenario, participants were asked the following question: “Please select the response that best describes the requirements for participating in this study.” Then, they were prompted to select the correct response from a list of five possible scenarios.

### Hypotheses and Data Analysis

The following hypotheses focused on the two factors of study and were generated before data collection.

#### Hypothesis 1

There will be a main effect of the number of device requirements factor on all mHealth acceptability dimensions, such that requiring participants to only use a single mobile device, as opposed to requiring multiple devices, will elicit higher levels of a priori acceptability for mHealth intervention participation across seven acceptability dimensions (ie, perceived interest, benefit, utility, enjoyment, confidence, difficulty in participating, and perceived likelihood of participating).

#### Hypothesis 2

There will be a main effect of the tailoring factor on all mHealth acceptability dimensions, such that providing tailored personalized content to participants, as opposed to generic content, will elicit higher levels of a priori acceptability for mHealth intervention participation across seven acceptability dimensions (ie, perceived interest, benefit, utility, enjoyment, confidence, difficulty in participating, and perceived likelihood of participating).

Exploratory analyses were conducted to examine if there were any significant interaction effects between the different levels of the two factors (ie, tailoring vs generic and single vs multiple devices) and acceptability ratings. Given the lack of previous evidence or strong theory on this issue, no hypotheses regarding interaction effects were made before the study design, data collection, and analysis.

Descriptive statistics were used to evaluate the sample characteristics and the careless responder items. SAS 9.4 (English) data analytic software was used to conduct analyses of variance to evaluate the effect of each hypothetical design scenario on the dimensions of a priori acceptability as well as the overall perceived likelihood of participating in the study. SAS’s PROC GLM procedure was used to generate models of the main effects of tailoring and device factors on acceptability dimensions as well as explore any possible interaction effects. No other factors (besides those indicated in the hypotheses and in the description of the exploratory analyses) were included in these models.

## Results

### Sample Characteristics

A diverse US sample (N=255) of English-speaking adults aged >18 years (female: 104/255, 40.8%; White: 208/255, 81.6%; aged 18-74 years) was recruited (Table 1). 

**Table 1:** Sample characteristics (N=255).

Characteristics		Values
**Sex, n (%)**		
	Female	104 (40.8)
	Male	155 (59.2)
**Race, n (%)**		
	People of color	47 (18.4)
	White	208 (81.6)
**Ethnicity, n (%)**		
	Hispanic or Latino	15 (5.9)
	Non-Hispanic or non-Latino	240 (94.1)
**Age (years)**		
	Range	18-74
**Family income (US $), n (%)**		
	10,000-40,000	107 (41.9)
	40,000-100,000	130 (50.9)
	>100,000	18 (7.1)
**Education, n (%)**		
	No Bachelor’s degree	128 (50.2)
	Bachelor’s degree	117 (45.9)
	Master’s degree	10 (3.9)

### Study Length and Careless Responder Results

Data collection for all participants was completed within 9 days. Participants answered 95.52% (1218/1275) of the careless responder questions correctly (Table 2).

**Table 2:** Careless responder question results (N=255).

Questions		Participants, n (%)
**Question 1: learning module**		
	Correct	249 (97.6)
	Incorrect	6 (2.4)
**Question 2: study scenario 1**		
	Correct	249 (97.6)
	Incorrect	6 (2.4)
**Question 3: study scenario 2**		
	Correct	235 (92.2)
	Incorrect	20 (7.8)
**Question 4: study scenario 3**		
	Correct	247 (96.9)
	Incorrect	6 (2.4)
**Question 5: study scenario 4**		
	Correct	238 (93.3)
	Incorrect	17 (6.7)
Overall correct careless responder questions (n=1275)		1218 (95.52)

### Effect of Design Factors on Dimensions of Acceptability

Table 3 provides the main effect results, and Table 4 provides the mean ratings across dimensions of acceptability. There were strong main effects of requiring multiple (ie, 2) devices on reports of the dimensions of acceptability. Requiring multiple devices (vs a single device) led to higher interest in the study (*P*=.008), more perceived benefit (*P*=.04), and greater anticipated utility (*P*=.004) but also lower confidence in performing study requirements (*P*<.001) and significantly higher perceived study difficulty associated with participation (*P*<.001). Requiring multiple devices was unrelated to perceived enjoyment (*P*=.59). Finally, requiring multiple devices was related to a lower likelihood of anticipated study participation (*P*=.02). 

**Table 3:** Main effects of the number of devices and tailoring on dimensions of acceptability.

Acceptability dimension	Main effect (devices)^a^				Main effect (tailoring)^a^		
	Mean square	*F* test (*df*)	*P* value	Mean square		*F* test (*df*)	*P* value
Interest	8.99	7.02 (1)	.008	198.43		154.85 (1)	<.001
Benefit	4.58	4.16 (1)	.04	120.27		109.33 (1)	<.001
Usefulness	9.33	8.30 (1)	.004	163.43		145.43 (1)	<.001
Enjoyment	0.35	0.30 (1)	.59	132.34		111.70 (1)	<.001
Confidence	47.26	40.69 (1)	<.001	17.06		14.69 (1)	<.001
Difficulty	100.06	81.93 (1)	<.001	1.57		1.28 (1)	.26
Likelihood to participate	5305.45	5.83 (1)	.02	58,210.97		63.96 (1)	<.001

^a^The manipulations of tailoring and the number of devices across dimensions of acceptability did not reveal any significant interaction effects.

**Table 4:** Means across dimensions of acceptability across each cell of the 2 (number of devices)×2 (tailoring) factorial design.

Acceptability dimension and tailoring		Devices, n	
		Single, mean^a^ (SD)	Two, mean (SD)
**Interest**			
	Generic	2.77 (1.13)	3.02 (1.23)
	Tailored	3.71 (1.05)	3.85 (1.12)
**Benefit**			
	Generic	3.22 (1.10)	3.45 (1.13)
	Tailored	4.00 (0.95)	4.05 (1.01)
**Usefulness**			
	Generic	3.09 (1.10)	3.37 (1.16)
	Tailored	3.98 (1.00)	4.10 (0.97)
**Enjoyment**			
	Generic	2.78 (1.05)	2.82 (1.14)
	Tailored	3.58 (0.98)	3.47 (1.17)
**Confidence**			
	Generic	4.15 (0.99)	3.72 (1.29)
	Tailored	4.41 (0.85)	3.98 (1.12)
**Difficulty**			
	Generic	1.84 (1.06)	2.52 (1.23)
	Tailored	1.81 (1.04)	2.39 (1.08)
**Likelihood to participate**			
	Generic	53.73 (30.18)	51.65 (32.19)
	Tailored	71.40 (27.32)	64.55 (30.79)

^a^All mean ratings are on a 1-5 scale (1=not; 5=very) except for likelihood to participate, which is on a 1-100 scale.

Similarly, there was a robust main effect of tailoring on acceptability, such that interest in the study, perceived benefit, enjoyment, confidence in performing study activities, perceived utility, and overall likelihood of participating in the study were each rated as significantly higher for studies featuring tailored versus generic content (all *P*<.001); in contrast, tailoring had no effect on ratings of perceived difficulty (*P*=.26).

There were no significant interactions between requiring multiple devices and providing tailored content noted for interest (*P*=.43), benefit (*P*=.17), enjoyment (*P*=.26), confidence (*P*=.94), utility (*P*=.20), and difficulty (*P*=.47). Similarly, the interaction of tailored content and requiring multiple devices was not related to the reported overall likelihood of participation (*P*=.21).

## Discussion

### Principal Findings

The overarching goal of this study was to examine if design factors influence a priori acceptability for participating in mHealth interventions and if this factorial- and vignette-based experimental design approach may be a useful method for examining such factors. More specifically, the study focused on evaluating how two common mobile intervention design elements—requiring multiple devices and providing tailored intervention content—affect a priori participant acceptability for participating in behavior change programs via mobile technology. Given its focus on how thoughts and beliefs may influence behavior, the TPB was used as a theoretical basis for conceptualizing how design decisions may predict acceptability (and subsequent intervention engagement) and informed this study’s hypotheses. Overall, the results of this study suggest that potential research participants are sensitive to mHealth study design components and participation requirements.

To date, the limited mHealth implementation research that has focused on mobile design and acceptability has been mostly observational and had participants respond to a particular array of features [12,25,26]. In contrast, this study experimentally manipulated two representative features and obtained ratings on each, thus combining the strengths of experimental and within-person designs. A risk associated with this approach was that viewing multiple arrays of design features (represented in the four vignettes or cells) would lead to order or conditioning effects (eg, some preference for the earlier or later vignettes presented or a carryover effect from one vignette to the next). To minimize this concern, this study presented four design scenarios in random sequences for all participants.

Multiple types of thoughts, some of which are inherently positive (eg, thoughts about beneficial experiences like enjoyment) and negative (eg, thoughts about burdensome experiences like difficulty) can combine to form larger overall beliefs about acceptability. This study focused on this by taking a multidimensional approach to the evaluation of multiple psychological constructs that may be at play when forming perceptions of acceptability. The goal of this study was to evaluate the directionality of these relationships and better understand which elements of mobile design factors (ie, content and number of devices) predict multiple dimensions of a priori acceptability, positively or negatively, in the context of being presented an opportunity to join an mHealth intervention.

### Hypotheses

Findings related to the first hypothesis (ie, requiring participants to use multiple mobile devices, as opposed to a single device, would elicit lower levels of a priori acceptability for participating in mHealth) were mixed. Intervention scenarios requiring multiple devices had clear and somewhat unexpected main effects on increasing (as opposed to lowering) perceived interest, benefit, and utility. In line with predictions, the results indicate that mHealth intervention scenarios that require multiple devices increase the perceived burden of participating, as they elicit higher levels of perceived difficulty and lower levels of confidence in fulfilling program requirements and lead to a lower reported overall likelihood of participating. There was also no main effect of requiring multiple- versus single-device requirements on anticipated enjoyment.

Why did the requirement of multiple devices lead to increased ratings on some dimensions of acceptability (ie, interest, benefit, and utility)? Perhaps participants generally view interventions with multiple devices as superior; perhaps such studies are seen as more scientific, legitimate, or reflect more innovative and potentially useful approaches to tracking and addressing behavior change. If present, such effects may have possibly been enhanced by the within-person factorial design; that is, as each person saw all the vignettes, it may have implicitly led to more direct contrasts between the design features (and led to a *more devices must be better* evaluation). Other dimensions of acceptability were in accordance with predictions, and common sense, in that participants appeared appropriately sensitive to the additional requirements of caring for and managing multiple devices over time (eg, charging smartphone batteries frequently and taking off wearable sensors to bathe and then replacing them) as functional barriers to joining an mHealth study. This is similar to previous posteriori research that suggests that requiring participants to use multiple devices over the course of a program may reduce acceptability [32]. More contextual data (eg, qualitative feedback) in future studies may be helpful to understand the effect requiring multiple devices, compared with a single device, has on a priori mHealth acceptability.

Findings related to the second hypothesis (ie, providing tailored content to participants, as opposed to generic content, would elicit higher levels of a priori acceptability for participating in mHealth) were largely supported by these results. Intervention scenarios featuring tailored content had strong main effects for enhancing perceived interest, benefit, enjoyment, utility, and higher intended participation compared with interventions providing only generic content. Research suggests that tailored content may be perceived by participants as having enhanced value (compared with generic content) because it can provide information specific to their individual health behavior needs [1,33,34]. Tenets of the TPB can be used to interpret these findings, such that designing mHealth interventions to provide tailored content to individuals (as opposed to generic messaging) may elicit personally beneficial thoughts and beliefs (ie, high acceptability) about participating. This in turn may enhance prospective participant motivation for signing up for mHealth studies featuring tailored content, which may translate to a higher likelihood of engaging with behavior change delivered via mobile technology. Although plausible, future research is needed to determine if high acceptability for tailored interventions at baseline before the intervention (a priori) actually translates to actual persistence and effective adoption of behavior change strategies throughout mHealth study participation.

There was no main effect of tailored content on perceived difficulty. This suggests that providing personalized versus generic content does not affect perceptions of effort or burden for participating in mHealth research. The hypothetical manipulation of tailoring in this study was a requirement for reading intervention messaging that is either tailored or generic. It is reasonable that participants do not perceive such tasks as being differentially burdensome, as there were no meaningful differences (eg, in length) between the two conditions. It is also possible, however, that the learning module did not provide enough information about additional requirements that sometimes accompany participation in tailored mHealth interventions. For example, these interventions may entail components such as active tracking, additional app installations, and syncing devices. These (and similar) tasks may increase the perceived burden of participating in tailored mHealth programs, and future studies may provide more specificity about such requirements to further investigate how tailoring may affect perceived difficulty.

There were no significant interaction effects between requiring multiple devices and providing tailored content noted for any of the dimensions of acceptability. Given the limited past research examining these two specific design factors together in an experimental fashion, this was an exploratory hypothesis. The results suggest that participants’ perceptions of acceptability for multiple devices and tailoring factors are not contingent on the presence of one factor or the other. Although no evidence was found about these two design elements in particular interacting with one another, perhaps other design features (or more extreme versions of these features; eg, carrying 4 devices) may show interactions. As such, this is seen as an important open issue for future mHealth design research, and additional theoretical considerations (eg, what elements of design features and their implications are thought to interrelate) and empirical evidence (eg, additional features, combinations, and levels) should be considered.

### Limitations and Future Directions

There are notable limitations to this study. Amazon’s web-based MTurk platform was used for the identification, enrollment, and compensation of research participants. MTurk served as a rapid recruitment service (N=255 participants within 9 days). It afforded access to a diverse sample of respondents (female: 104/255, 40.8%; White: 208/255, 81.6%; aged 18-74 years), and our findings (ie, careless responder items answered correctly: 1218/1275, 95.52%) suggest high data quality in this web-based experiment. In recent years, MTurk has been increasingly used in social and behavioral science research because of its ability to rapidly recruit and compensate large pools of research subjects [38]. However, there is also an ongoing debate as to whether research findings from MTurk participant pools are generalizable to the rest of the US population [39,40]. One issue is that samples recruited from MTurk generally exhibit higher educational attainment than the actual US population (ie, 35% of the US population possesses bachelor’s degrees as of 2018 [41]). This study followed a similar suit, as 49.8% (127/255) of the sample recruited via MTurk possessed a bachelor degree or higher.

This study investigated hypothetical mHealth design scenarios using web-based vignettes. This was useful for examining how design decisions may affect a priori (ie, prospective) acceptability, which may be related to self-selection into mHealth research or motivation to fulfill intervention requirements. Given the hypothetical nature of the vignettes and a priori design, one limitation is that this study did not allow for examination of acceptability posteriori (ie, after participants actually engage with a real mHealth intervention in the field). It is unclear how acceptability (even if it is high before engagement) changes over time and whether differences in design factors affect posteriori acceptability in a similar direction as a priori acceptability. For example, perhaps participants are highly motivated to participate in an intervention that provides tailored content, but over time, their acceptance and motivation may decline. Real-world engagement with mHealth behavior change programs may also enhance acceptability above and beyond baseline measures. In addition, because of the hypothetical nature of the study vignettes, these findings do not directly provide information about how a priori acceptability is associated with other measurable implementation outcomes (eg, actual adherence and sustained engagement). Longitudinal experimental designs within randomized controlled mHealth trials (eg, as opposed to hypothetical web-based study scenarios, as used here) are needed to better understand these relationships. A quasi-experimental study of mHealth design and acceptability (ie, manipulating mHealth design features and assessing both a priori and posteriori acceptability) may also help understand how mHealth acceptability may change over time between different design conditions. Each of these are important questions that could be examined in future real-world mHealth implementation research.

Another limitation to the design of this study was that participant health was not assessed. As such, it is unclear whether the sample was at risk of behavioral health challenges. This makes further generalizability of these findings to patient populations with health behavior challenges unclear, as there may be reliable differences across meaningful subsamples (eg, sick individuals may be more highly motivated to pursue treatment and thus more *tolerant* of high burden design features). Future mHealth implementation studies (including those conducted via web-based surveys) should screen and select participants to ensure that the findings are applicable to the target population of interest. Another approach would be to consider enrolling healthy controls to match patient participant groups to investigate whether differences in mHealth acceptability exist depending on the saliency of health behavior change needs.

Finally, to convey the meaning of generic versus tailored content delivered via mHealth to support behavior change, participants were provided with a relatable example of a behavior to which this messaging could be applied. Physical activity (in the form of *step count*) was used as an exemplar health behavior to describe differences between generic and tailored messaging. This behavior was selected under the assumption that most participants would likely be at least vaguely familiar with the concept of tracking activity, especially given increasing trends and publicity worldwide for wearing fitness trackers (eg, Fitbit) and smartwatches (eg, Apple Watch) to monitor health [42]. However, physical activity is a complex health behavior, and it is possible that participants perceived activity-related behaviors as more difficult to change compared with other familiar health behaviors (eg, medication adherence and applying sunscreen). Acceptability ratings and the impact of design factors may be differentially related across different behaviors, and the features and characteristics of these behaviors that may drive any divergent associations is an important topic for future consideration. More generally, it is unclear which behavior change outcomes mHealth is best suited to address to support buy-in and engagement in target populations (eg, Is an mHealth intervention targeting physical activity more or less acceptable than one focused on healthy eating?). Some research has shown disparities in participant engagement with mHealth-based behavior change interventions based on demographic moderators (eg, age and health literacy) [43], and such differences between participants may also play a role in perceptions of acceptability for supporting different types of behavior change with technology. Future research should further evaluate how acceptability for participating in mHealth may change differentially depending on the intervention’s target behaviors, as well as explore other possible moderators inherent to target health behaviors that may influence this relationship (eg, frequency of behavior, intensity of behavior, and perceived behavioral norms).

### Conclusions

This study showed that differences in intervention design factors, specifically the number of devices required and tailoring, affect various dimensions of participant acceptability for engaging in behavior change programming via mHealth. Some limited previous work examining acceptability for these design factors was conducted posteriori (and studied these factors separately); however, understanding a priori perceptions is advantageous for designing mobile interventions that support up-front buy-in and acceptability from individuals for participating in mHealth research. Previous implementation research and theoretical models from behavioral science suggest that acceptability is a predictor of the uptake and utilization of behavior change interventions. This study supports the hypothesis that participants are sensitive to the mHealth design decisions made by researchers, specifically those related to the provision of the number of devices required for participation and tailored content. The results show that requiring participants to use multiple devices increases perceived burden and reduces their overall reported likelihood of participating and that providing participants with tailoring in mobile behavior change interventions is beneficial for enhancing a priori acceptability.

This work also contributes to the early development of evidence-based recommendations in mHealth design to support participant acceptability. More generally, this method serves as proof of concept for how other design features can be tested—singly or in combinations of factors—and using assessments of a priori acceptability, posteriori acceptability, or both. As the need for investigating mHealth implementation factors continues to emerge, researchers in this space may also consider MTurk as an inexpensive and effective recruitment tool with a wide reach and reliable, attentive respondents in cases where unique sample characteristics are not essential. Future studies on the relationship between mHealth design elements and participant motivation to engage with mHealth should further explore multiple dimensions of acceptability experimentally in controlled trials in both clinical and community samples. Overall, this will afford more reliable data on the effects these common mHealth design elements have on influential mHealth implementation factors (eg, not just a priori acceptability, but also posteriori acceptability, real-world compliance, and adherence).

## Abbreviations

HIT: human interaction task
mHealth: mobile health
MTurk: Mechanical Turk
TPB: theory of planned behavior
